# Barriers and opportunities to improve fluid balance recognition and reduction: a qualitative study

**DOI:** 10.3389/fped.2026.1830086

**Published:** 2026-06-11

**Authors:** Chloe Braun, Celeste G. Dixon, Ami J. Shah, Adam Dziorny, Julie C. Fitzgerald, Susan Martin, James Odum, Melissa Ryan, Samantha Whitfield, Lori B. Bateman, Denise C. Hasson

**Affiliations:** 1 Department of Pediatrics, University of Louisville School of Medicine, Louisville, KY, United States; 2 Department of Anesthesiology & Critical Care Medicine, Children’s Hospital of Philadelphia, University of Pennsylvania Perelman School of Medicine, Philadelphia, PA, United States; 3 Department of Pediatrics, Hassenfeld Children’s Hospital at NYU Langone Health, New York, NY, United States; 4 Department of Pediatrics, University of Rochester School of Medicine, Rochester, NY, United States; 5 Department of Pediatrics, University of Alabama, Birmingham Heersink School of Medicine, Birmingham, AL, United States; 6 Department of Medicine, University of Alabama, Birmingham Heersink School of Medicine, Birmingham, AL, United States

**Keywords:** critical care, fluid accumulation, fluid overload, pediatrics, semi-structured interviews, volume overload

## Abstract

**Introduction:**

Excess cumulative fluid balance is a common complication impacting critically ill pediatric patients that is associated with worse clinical outcomes. Nevertheless, there are inconsistent practices in recognizing and reducing excess fluid balance.

**Purpose:**

Assess contextual factors that inform fluid-based practices across four pediatric intensive care units.

**Methods:**

We conducted semi-structured interviews across 4 PICUs from August to October 2025. Through an iterative process informed by prior survey data, we followed the established implementation science Reach, Effectiveness, Adoption, Implementation, Maintenance/Practical Robust Implementation and Sustainability Model framework to develop an interview guide. Questions explored fluid balance recognition and reduction. Participants were recruited using purposive sampling via email recruitment. We used Rapid Assessment Procedures with two independent coders. Thematic saturation was reached within the first round of interviews.

**Results:**

We performed thirty-eight interviews comprised of nurses (*n* = 19), physicians (*n* = 12), advanced practice providers (*n* = 4), and trainees (*n* = 3). Institutions had balanced numbers of interviews. Theme 1 centered on the limitations of data used for fluid balance recognition, namely that these data are affected by patient variability, rely on subjective exam findings, and depend on inaccurate tools. Theme 2 highlighted insufficient emphasis on limiting unnecessary fluid administration. Theme 3 addressed cultural barriers including educational gaps, lack of standardization, entrenched unit practices, and insufficient communication.

**Discussion:**

These themes highlight the need for accurate data reporting, shifts in emphasis of fluid management, and cultural/systemic changes. We theorize that these suboptimal approaches to fluid balance management could inform clinical decision support tools, research priorities, and quality improvement initiatives.

## 1 Introduction

Excess cumulative fluid balance (CFB, an objective measure of fluid status overtime) is associated with increased morbidity and mortality among critically ill pediatric patients and has been termed fluid accumulation (1, 2). Despite known detrimental impacts on outcomes, fluid accumulation remains a common and underrecognized phenomenon (3, 4). While prevention, early recognition, and early intervention are possible approaches to limit fluid accumulation, timing and approach to interventions are not standardized and impact on patient centered outcomes is not well elucidated.

Accurate assessment of fluid balance depends on two primary data elements: patient weight and documented intake and output. However, these foundational measures are often inconsistent and prone to inaccuracy. As a result, even the basic calculation and interpretation of CFB can be challenging, complicating efforts to prevent, recognize, and intervene (5, 6). Given the complexity of contributing factors, there remains no broadly applicable consensus on when excess CFB becomes pathologic, when it should be intervened upon, or what interventional approach should be taken.

Therefore, we set out to assess contextual factors that inform decisions around CFB management at four pediatric intensive care units (PICU) across the United States— specifically related to CFB recognition and CFB reduction. We theorized that novel themes would emerge unique to individual institutions, that would provide new insights into ways we can improve our recognition, prevention, and treatment of excess CFB.

## 2 Methods

### 2.1 Study design

This is a qualitative descriptive study utilizing semi-structured in-depth individual interviews to assess contextual factors that inform fluid-based practices across four PICUs. Institution specific descriptions and patient demographics were determined from our group's prior work (7) (Supplementary Table S1). For our analysis, we opted for action oriented Rapid Assessment Procedures (8), as our goal was to derive real world practical data that could be readily implemented in a timely fashion. This study was approved by the Institutional Review Board at each respective institution (300014896, 00010554, 25-00596, 25-023567) with waiver of written consent. Verbal consent was received by all participants at the beginning of the interview. We utilized the Standards for Reporting Qualitative Research (9). (Supplementary Document S1).

### 2.2 Purposive sampling

We aimed to capture the multidisciplinary nature of the PICU utilizing a purposive sampling approach, by recruiting nurses, advanced practice providers, physician trainees (i.e., residents who completed their PICU rotations, fellows), and attending physicians that practice in a PICU setting via listserv emails. To reach goal recruitment, a follow-up email or a flyer posted in the PICU was used. We chose participants on a first come-first serve basis regardless of participants' role.

### 2.3 Interview guide development

Through an iterative process informed by prior survey data (7), we followed the well-established, implementation science based RE-AIM/PRISM framework (Reach, Effectiveness, Adoption, Implementation, Maintenance/Practical Robust Implementation and Sustainability Model) (10) to develop a single interview guide used across institutions to explore barriers and facilitators behind CFB recognition and reduction (Supplementary Document S2). The interview guide was written and edited by members of the research team (4 PICU attendings), reviewed by the analysis team (researchers with masters in Public Health or Science in Healthcare Quality and Safety), and subsequently approved by all members of the collaborative. This interview guide was piloted 3 times at separate institutions after which small wording changes were made for improved clarity. Additionally, certain questions were highlighted to prioritize if time in interview became limited. Because thematic saturation was reached during the initial analysis, no modifications were made to the interview guide/process in response to evolving findings.

### 2.4 Interview process

We conducted semi-structured interviews across four PICUs from August to October 2025. Interviews were performed by one of six researchers who were trained on how to lead semi-structured interviews (DH, AS, AD, RJ, CB, and CD). The interviewers have known interest in CFB topic and may or may not work with the participants clinically. We utilized both virtual and in-person interview techniques, based on participants' preference. Interviews were performed via virtual meeting (Zoom Video Communications, San Jose, CA or Microsoft Teams, Washington DC) on institution-specific virtual communication platforms. If preferred by the participant, in-person interviews took place at the respective hospitals either in or near the PICU in a quiet, private area.

Recording and transcription was performed via video recording/automatic transcription via Zoom or Microsoft Teams software, audio recorder applications (Rev, Austin, TX), or digital recorder (Sony ICD-PX470). Manual editing by the interviewer was performed to ensure accuracy and deidentification. Depending on the institution (and thus feasibility of offering compensation), some participants were offered a nominal monetary amount via gift card for completing the interview.

### 2.5 Demographic data collection

We collected baseline demographic information from each participant prior to the interview via a standardized REDCap survey (11), including primary unit type, professional background, subspecialty experience, number of years in practice, and any experience at another institution. Data for each center's participants were collected in their own respective REDCap environments, then deidentified prior to transfer to a single institution (UAB) for aggregation and analysis.

### 2.6 Data processing and analysis

We utilized two independent coders (SW and MR) to ensure accuracy and limit potential bias. Thematic saturation was defined as the point at which no new themes or subthemes emerged during analysis (12). Analysis began with transcript familiarization (note taking and developing a better understanding of the CFB topic). Results were then grouped broadly by similar themes through an iterative process. SW and MR then met to discuss, compare notes, and consolidate themes. Representative quotes were identified using agreed upon themes. Subsequently, themes were compared across institutions and outliers (institution, role, individual interviews) were identified. Preliminary results were reviewed by the research team and discussed with the coders. Researchers then reviewed the themes to highlight interventions discussed by participants.

## 3 Results

Thirty-eight interviews (32 virtual, 6 in person) were conducted across four institutions (Table 1). Institution A, B, C, and D had balanced numbers of interviews: 7, 10, 9, and 12 respectively. The participants included PICU nurses (*n* = 19, 50%), PICU physicians (*n* = 12, 31%), advanced practice providers (*n* = 4, 11%), and physician trainees (*n* = 3, 8%) with the majority practicing in a medical/surgical PICU. Fifteen participants (40%) had experience practicing at more than one institution. Just over half of participants reported 0–5 years of experience (*N* = 20, 53%), and 11 (29%) participants had >10 years' experience. Thematic saturation was achieved after 23 interviews; however, all 38 interviews were analyzed to ensure comprehensive representation across roles and institutions.

**Table 1:** Demographic information of participants.

	Institution A	Institution B	Institution C	Institution D	Combined (%)
Number of interviewees	7	10	9	12	38
Nurse	5	6	5	3	19 (50%)
Advanced practice provider	0	1	0	3	4 (11%)
Attending Physician	2	1	3	6	12 (31%)
Fellow Physician	0	0	1	0	1 (3%)
Resident Physician	0	2	0	0	2 (5%)
Years of experience					
0–5	4	5	5	6	20 (53%)
6–10	0	2	3	2	7 (18%)
11–15	3	1	0	2	6 (16%)
>15	0	2	1	2	5 (13%)
Primary unit type					
Medical/surgical PICU	7	6	9	10	32 (84%)
Cardiac ICU	0	3	0	0	3 (8%)
Other	0	1	0	2	3 (8%)
No. with experience at >1 institution	4	1	4	6	15 (40%)

**Figure 1:**
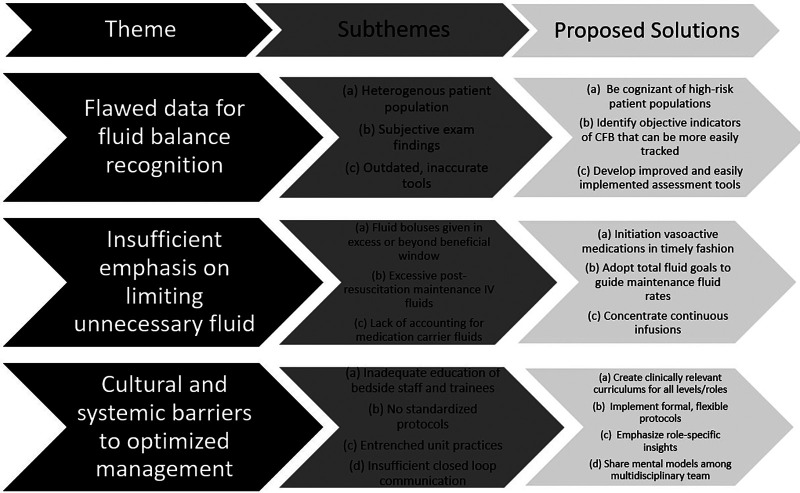
Themes, subthemes, and proposed solutions.

Three themes and 10 subthemes emerged. With each subtheme, proposed interventions or areas of future research were gleaned from participants' direct responses. These are summarized in Figure 1.

### 3.1 Theme 1: data used for fluid balance recognition are flawed

Nearly all interviewees stated they relied on subjective physical exam findings and more objective intake/outputs and weights recorded in the electronic health record (EHR) to recognize a patient's CFB. However, many interviewees pointed out reasons that these data are flawed, listing factors that contribute to erroneous or incomplete information that are then used to calculate and thus recognize a patient's CFB.

#### 3.1.1 Data are impacted by patient variability

PICU patients encompass a wide range of demographics and are afflicted by heterogeneous pathophysiologic processes. For example: identifying CFB changes in a 3-kilogram neonate with sepsis requires a different approach than a dehydrated 18-year-old with diabetic ketoacidosis. One participant specifically noted that physical exam findings may be more difficult to interpret in patients with darker skin tones, highlighting limitations of traditional exam markers. This subtheme described how different patient factors impact CFB recognition.

- “It is really hard to assess fluid status in morbidly obese patients.” (Attending Physician)
- “I think post-surgical cardiac patients are also tricky depending on the repair or sometimes before a repair also because of their hemodynamics.” (Attending Physician)
- “I think it's much harder to tell skin changes, such as mucous membranes and the [capillary] refill in children with darker skin color.” (Nurse)

The proposed intervention for this theme is understanding that patient variability is nonmodifiable, highlighting factors that make patient groups higher risk, and implementing a system whereby higher risk patients receive focused management.

#### 3.1.2 Data rely on subjective physical exam findings

This subtheme acknowledges that the physical exam remains cornerstone in pediatric critical care. Physical exam findings are provider dependent and are subject to provider's level of experience and implicit and explicit biases. Additionally, a patient's family member may have an incongruous perception of the patient's appearance compared to a medical provider.

- “Looking at the patient and see if they seem really puffy and edematous, I look at that as a fluid balance indicator.” (Nurse)
- “Yeah, I think physical exam would be the most important for me. So, looking at peripheries, capillary refill time, and pulse volume, but as well those other things like oxygenation requirements, weight and fluid balance, daily weights, monitoring their ins and outs and what that balance is.” (Attending Physician)
- “I try to not use just one variable … it's weight changes, it is fluid balance from an I and O's standpoint … laboratory values … and then there's physical exam.” (Attending Physician)

As these quotes illustrate, a proposed solution to the subjectivity of physical exam is taking physical exams in context with other potentially more objective data points.

#### 3.1.3 Data depend on outdated or inaccurate tools

Patient weights and intake/output trends form the foundation of calculating a patient's CFB. Participants described multiple vulnerabilities in these measures as summarized in Table 2. Additionally, novel diagnostic tools are lacking in this domain.

- “I put all of those data points together, because I don't really have great confidence in any single one of them.” (Trainee Physician)
- “Nobody seems to use the same scales. Nobody knows what a bed is zeroed with.” (Nurse)

**Table 2:** Causes of inaccurate CFB data.

Intake and output	Weights
Medication pump integration errors	Dysfunctional scales
Incomplete records from other locations (ex. Outside hospital, operating room, emergency room)	Patient mobility limiting weighing options and complicating bed weights
Missed medication flushes	Erroneous admission or anchor weight
Wound drainage inconsistently captured	Inconsistent scales being used between units
Outputs unable to be accounted for (e.g., emesis, urine, stool)	Inconsistent bed zeroing techniques
Excrement discarded by patient/family prior to measurement	Safety concerns of moving critically ill patients
Timing of nursing documentation does not match timing of provider chart checking for rounds	Time constraints of busy units (weighing frequently requires multiple staff members)
High acuity resuscitations prevent timely documentation	Daily weights not consistently ordered
Insensible losses not captured	Impact of medical devices (ex. EEG leads, ECMO catheters, wound closure devices)
Medications overfill volume not documented	

EEG, Electroencephalogram; ECMO, extracorporeal membrane oxygenation.

Current methods and tools, despite best efforts and increased awareness, will likely always have inaccuracies. For this reason, a proposed approach to improvement includes innovative research and development of improved assessment tools.

### 3.2 Theme 2: there is insufficient emphasis on limiting unnecessary fluid administration

When asked how the medical team can reduce CFB, the predominant theme focused on ways inadvertent and discretionary fluids are administered. These fluids are administered both during initial resuscitation as well as the stabilization phase of illness. Participants discussed not only fluids intentionally prescribed (e.g., maintenance fluids) but also fluids the providers may not realize are being given (e.g., flushes, overfilled intravenous fluid bags).

#### 3.2.1 Resuscitative fluids may be used in excess

At times, resuscitative fluid administration (e.g., fluid boluses, blood products) is necessary and lifesaving. But this form of fluid administration may continue when it no longer accomplishes the goal of increasing a patient's cardiac output.

- “I feel like we do give too much fluid sometimes … we have providers who will drag their feet on starting pressor support.” (Nurse)

A proposed intervention to limit inappropriate resuscitative fluids is to stop fluid bolus administration and/or initiate vasoactive medications at an earlier time point, particularly when a patient is no longer fluid responsive.

#### 3.2.2 Maintenance fluids may be used in excess

Similar to resuscitative fluids, maintenance fluids (i.e., fluids given continuously, typically containing dextrose and electrolytes) can be necessary. In certain patients, they can prevent hypoglycemia and dehydration and provide needed electrolyte administration. Nevertheless, maintenance fluids may not always be necessary, may not account for other fluid administration, and contribute to cumulative fluid burden. Although originally intended for patients unable to tolerate enteral intake, they are sometimes continued in patients with adequate gastrointestinal function.

- “If a kid has a G tube and is here for whatever reason, but we automatically put them on fluids, it's like, do they actually need to be on fluids, or can they just be on their regular home feeds?” (Nurse)
- “Getting them to enteral [feeds] as quickly as possible is kind of first and foremost for me.” (Attending Physician)

Proposed interventions stem from being more intentional about maintenance fluid prescription including earlier conversion from IV fluids to enteral nutrition. Additionally, patients should have a prescribed total fluid goal such that maintenance fluids would be adjusted for the volume of other administered fluids.

#### 3.2.3 Carrier volumes may be used in excess

Fluids are necessary to ensure that medications are delivered at an appropriate rate and to clear whatever tubing through which medications are given. This can be in the form of flushes after intermittent medications or in a continuous fashion to keep vascular access points open or continuous medications reliably delivered. These volumes are administered in higher than necessary amounts and are inconsistently standardized and charted.

- “Looking at the concentrations of meds… maybe it's a patient that weighs a lot more, so having a certain concentration may mean putting on twice the amount of fluid.” (Nurse)
- “And then I think we underestimate the amount of volume we give patients through their medications and in terms of flushes and it actually adds up to a pretty significant amount, and I think we don't probably take that into account enough. I think I've definitely been surprised when I really sat and looked, OK, let's add it all up.” (Attending Physician)
- “All of our patients across the board, it's three [mL/h], and if they have a triple-lumen catheter, forget about it. That's 9 [mL/h].” (Nurse)

Proposed interventions include using more concentrated infusions when able, saline locking intravenous access points, and standardizing flush administration (both volume given and charting practices).

### 3.3 Theme 3: there are cultural and systemic barriers to optimized fluid balance management

This theme focused beyond the logistical barriers of CFB management, but rather on how the structure of medical training and practice in a high acuity academic setting influences patient care. This is complicated by the fact that the multidisciplinary medical team is made up of members from diverse educational backgrounds with frequent staffing (and intentional trainee) turnover. For example, resident physicians typically rotate through the PICU only several weeks at a time, may not receive formalized training on CFB management, and must quickly adjust to CFB practices that differ from other care areas.

#### 3.3.1 Education gaps related to CFB are ubiquitous

Members of the medical team may be unaware of the clinical significance of excess CFB. Lack of awareness makes recognition and intervention challenging, if not impossible. Inexperience driven by bedside nursing turnover and physician training structure lends itself to unintentional lapses in attention to CFB management.

- “I do want to say we get a lot of teaching in the PICU … but I don't think we ever did one on fluid balance.” (Attending Physician)
- “I think education is a huge one. Our unit's very young right now, and so … I think that being the case, we have young nurses teaching young nurses.” (Nurse)
- “The amount of times that I am working with a nurse who doesn't necessarily know that we have to update our I/Os every single hour … It's kind of shocking.” (Nurse)

Proposed interventions include targeted education or dedicated portions in orientation for inexperienced team members.

#### 3.3.2 Lack of standardization

This refers to standardization of all components of CFB management, from bedside logistics to provider practice. Confounded by lack of evidence in the medical literature, inconsistencies relate to EHR data entry, order entry, emphasis placed on fluid management, and thresholds for concern/action that are rarely shared with the whole medical team. Across all four institutions, there were no identified globally applied guidelines or protocols to inform fluid practices.

- “I guess it's frustrating when there are so many differences in practice and opinion. Even just what fluid are we going to bolus with … how some people are much more liberal with their volume resuscitation.” (Nurse)
- “I think just like anything, when you have some sort of standardization you run the risk of not looking at the whole patient.” (Attending Physician)

Proposed interventions include implementation of consistent, formal, and yet flexible protocols, while allowing variability when a patient's unique factors require alternative management. Similarly, with increased education, more consistency may naturally follow as perceived importance becomes shared.

#### 3.3.3 Entrenched unit priorities do not emphasize optimal CFB management

Presently, emphasis on CFB management is not universal. This may reveal itself as lack of emphasis on the importance of daily weights, infrequent calculation of CFB, and deeply engrained practices such as default to maintenance IV fluid prescription.

- “I think our medical training, honestly … if a kid is NPO, you put them on IV fluids. It's kind of the way.” (Attending Physician)
- “[calculating fluid balance] is actually not even part of our role or our scope … the lines are a little gray there.” (Nurse)
- “I don't think we stress the importance anymore of why we don't want to overload our patients.” (Nurse)

Proposed interventions include incorporating and valuing role-specific insights and utilizing the strengths of multidisciplinary approaches.

#### 3.3.4 There is insufficient communication between team members

Influenced by the multidisciplinary nature of PICU teams, a shared mental model is sometimes not clearly conveyed to all team members. More junior team members may not feel comfortable asking questions, when in reality the answers are often nuanced. Additionally, communication barriers and patient specific factors influence how and when concerns about CFB are discussed.

- “A lot of times the providers will have a goal fluid balance, and we don't ever really discuss, and then it changes every single shift.” (Nurse)
- “I think it should be discussed all the different things that we talked about … at least twice a day, maybe not every checkout rounds, but morning and night.” (Attending Physician)
- “If there was some sort of way on rounds that we could trigger that conversation, whether it being added to the nursing work list.” (Advanced Practice Provider)

Proposed interventions include standardizing the approach to CFB discussions (e.g., establishing a set time of day to formally check on CFB, or incorporating CFB goals into a note template, nursing report, or safety checklist) as well as establishing a shared mental model to each patient's CFB care.

## 4 Discussion

This project leverages expertise across multiple domains of pediatric critical care to establish an understanding of real-world context behind recognition and optimization of CFB management. Despite institutional differences in size, patient demographics, and geographic location, themes were relatively consistent across institutions. This study identified several contextual factors that may help improve management. Namely, there are easily addressed cultural and systemic barriers that may improve CFB management through education and improved communication. In addition to echoing what prior literature has shown, these interviews delineate next steps to optimizing patient care. Contrary to our initial theory, themes were relatively consistent across institutions, suggesting that barriers to fluid balance recognition have the potential to be more universal than institution specific.

Our first theme explored the constraints of the data used in CFB practice and highlighted patient variability, subjective exam findings, and inaccurate and outdated tools. While prior literature has shown weight-based determination of CFB and intake/output based are similar in predicting outcomes (13, 14), many questions remain, such as ideal weight to use in CFB calculation or what threshold should be used for intervention (15). While those questions are important, prior work and the themes identified with our interviews highlight the need for a more reliable and feasible approach to measuring and reporting weights and I/Os (6). Of note, our respondents did not discuss other assessments of fluid balance and fluid responsiveness such as central venous pressure monitoring, ultrasound assessments, or bedside assessments such as passive leg raise or pulse pressure variability. We cannot speculate if this is due to inaccessibility, lack of education, or simple underutilization. The importance of accurate measurements and related pitfalls (e.g., not capturing insensible losses) have been emphasized by the Pediatric Acute Disease Quality Initiative (15). Unsurprisingly, a recent systematic review of 23 studies evaluating quality of CFB monitoring concluded that CFB documentation is inaccurate (5). The heterogeneity of these studies limited the ability to perform a meta-analysis, further highlighting the need for ongoing research. Another large gap remains the lack of novel tools or technology to quantify CFB (16). Although the risks of pulmonary artery catheters were deemed to outweigh their benefits, few if any tools have been proposed to replace or supersede their ability to determine intravascular volume status (17).

Our second theme highlighted unnecessary fluid administration, a concept known as “fluid creep” (18). Our qualitative results mirror quantitative studies reporting that discretionary fluid can make up more than 60% of fluid administered on PICU day 1 (4), and that this discretionary fluid has been associated with higher mortality (18). One subtheme discussed appropriate limitation of resuscitative fluids and earlier initiation of vasoactive medications. As more data become available regarding safety of administering vasoactive medications peripherally or the benefit of earlier administration, this may become common practice (19–21). Determining the threshold when a patient is no longer physiologically fluid responsive can be challenging. Promising techniques like point of care ultrasound to measure inferior vena cava compressibility and venous excess ultrasound (VExUS) scores offer clinically relevant information on fluid status but also have limitations that impede their widespread incorporation into fluid resuscitation algorithms (22, 23). Another subtheme addressed excessive maintenance fluids. Published in 1957, the Holliday-Seager method remains universal for calculating maintenance fluid requirements (24) but there has been limited research recently in appropriate amount of maintenance fluid administration. A recent survey of providers in the European Society of Pediatric and Neonatal Intensive Care found 70% of respondents believe lack of guidelines and gaps in training lend themselves to suboptimal fluid management (25). While one proposed solution to these problems is setting a total fluid goal that incorporates all inputs, a recent quality improvement (QI) project revealed total fluid goals were infrequently ordered and inconsistently followed (26).

Our final theme discussed cultural/systemic barriers to optimal CFB management. Our participants identified educational gaps among all team members in addition to inconsistent approaches from attending providers. Nelson et al. identified that even among providers, awareness of morbidity and mortality associated with fluid accumulation was rare (3). Similarly, they found calculation and discussion of CFB was uncommon. As our participants identified, these inconsistencies lead to confusion and lack of standardization. Another prior survey of pediatric intensivists identified that 29% of providers do not calculate cumulative CFB (27). Our qualitative results suggest this could be higher in practice.

We identified numerous gaps in CFB management that are ripe for study and development. Many of the strategies that emerged during the interviews have been previously implemented in a separate single center QI initiative that developed a rounds' checklist (28). Although the authors found that their interventions decreased the prevalence of patients with cumulative CFB > 10%, it did not ultimately impact ICU outcomes (28). A prescriptive approach may be necessary, with tools to simplify and automate their proposed workflows. Clinical decision support tools within the EHR could assist with early identification and intervention. This requires further research to assess feasibility, adoption, and sustainability as well as larger studies to assess if these implementations improve patient-centered outcomes.

Two strengths of our approach increase the transferability of this work: the multi-institutional nature and number of interviews. Our work has some important limitations. Interviewers have an interest in the topic and though none had formal leadership positions, many have a perceived leadership role over participants, which could have introduced bias. We attempted to mitigate this with a pre-interview script emphasizing no right or wrong answers, that responses would stay anonymous, and that participants were not obligated to participate. To reduce perceived bias, data analysis was performed by a secondary team with no relationship to the participating PICUs and minimal domain expertise. Finally, due to the multi-institutional nature of our project, we had six interviewers which could have impacted consistency in interview administration. This was not only the most feasible approach, but also insight into the respective institutional practices likely enriched the semi-structured nature.

## 5 Conclusion

Consistent themes across institutions highlight the need for accurate data reporting, emphasis on the importance of CFB management, and cultural and systemic changes that focus on greater education and deliberate communication. We believe these identified gaps in CFB management have the potential to inform innovative technology, including implementation of novel tools, as well as research priorities and QI initiatives to advance CFB recognition and reduction among PICU patients.

## Data Availability

The original contributions presented in the study are included in the article/Supplementary Material, further inquiries can be directed to the corresponding author.
