# Supplementary material for: Barriers and opportunities to improve fluid balance recognition and reduction: a qualitative study
Source: Front Pediatr. 2026 Jun 11;14:1830086. doi: 10.3389/fped.2026.1830086 (PMC13294464; doi:10.3389/fped.2026.1830086)
Supplement: Supplementary file 2 [file Table1.docx]

Supplementary Table 1: Institution specific descriptions and patient demographics

|  | Institution A | Institution B | Institution C | Institution D |
| --- | --- | --- | --- | --- |
| Number PICU beds | 22 | 12 | 24 | 74 |
| Number cardiac ICU beds | Not included | 15 | Not included | 38 |
| Extracorporeal support capabilities | ECMO, RRT | ECMO, RRT | ECMO, RRT | ECMO, RRT |
| Level 1 Trauma Center | No | Yes | Yes | Yes |
| Unique ICU encounters, n^a^ | 509 | 388 | 690 | 1263 |
| Patient age, years  (Median, IQR) | 6.8  [2.0, 13.8] | 4.2  [1.0, 12.5] | 7.4  [2.0, 14.5] | 3.6  [0.8, 10.5] |
| Race, n (%) ^b^ | | | | |
| White or Caucasian | 332 (60%) | 226 (53%) | 421 (57%) | 627 (46%) |
| Black or African American | 87 (16%) | 68 (16%) | 257 (35%) | 288 (21%) |
| Asian | 36 (7%) | 5 (1%) | 2 (<1%) | 53 (4%) |
| Native Hawaiian or Pacific Islander | 5 (1%) | 0 (0%) | 0 (0%) | 1 (<1%) |
| American Indian or Alaskan Native | 4 (1%) | 0 (0%) | 2 (<1%) | 2 (<1%) |
| Other or Multiple Responses | 74 (13%) | 31 (7%) | 15 (2%) | 333 (25%) |
| Unknown | 15 (3%) | 97 (23%) | 38 (5%) | 52 (4%) |
| Ethnicity, n (%) ^b^ | | | | |
| Hispanic or Latino | 119 (22%) | 31 (7%) | 83 (11%) | 236 (17%) |
| Not Hispanic or Latino | 263 (48%) | 292 (68%) | 624 (85%) | 1046 (77%) |
| Other | 171 (31%) | 104 (24%) | 28 (4%) | 74 (5%) |
| ICU Length of stay, hours  (Median, IQR) | 45.7  [25.0, 78.9] | 43.7  [20.6, 106.4] | 35.0  [21.3, 69.7] | 54.8  [29.0, 118.3] |
| Hospital Disposition ^c^ | | | | |
| Home or Self Care | 477 (93.7%) | 362 (93.3%) | 648 (93.9%) | 1175 (93.0%) |
| Another Facility or Other | 28 (5.5%) | 15 (3.9%) | 18 (2.6%) | 64 (5.1%) |
| Expired | 4 (0.7%) | 11 (2.8%) | 24 (3.5%) | 24 (1.9%) |

^a^ Represents number of unique ICU encounters over 6-month period of time

^b^ Race and ethnicity are based on electronic health record extracted values and may not reflect patient self-reporting or be inclusive of all available categories.

^c^ Hospital disposition denominator is based on hospitalizations and not ICU admissions

ICU-Intensive care unit. IQR-interquartile range. RRT- renal replacement therapy, ECMO- extracorporeal membrane oxygenation
